# The relationship between peripheral neuropathy and efficacy in second-line chemotherapy for unresectable advanced gastric cancer: a prospective observational multicenter study protocol (IVY)

**DOI:** 10.1186/s12885-019-6163-6

**Published:** 2019-10-11

**Authors:** Hiroaki Tanioka, Takeshi Nagasaka, Futoshi Uno, Masafumi Inoue, Hiroyuki Okita, Yosuke Katata, Hiromitsu Kanzaki, Hidekazu Kuramochi, Hironaga Satake, Yoshiaki Shindo, Akira Doi, Jyunichiro Nasu, Haruhiro Yamashita, Yoshiyuki Yamaguchi

**Affiliations:** 1 Department of Clinical Oncology, Kawasaki Medical School Hospital, Kurashiki, Japan; 2 Department of Surgery, Okayama Rosai Hospital, Okayama, Japan; 3 Department of Gastroenterology, Okayama Red Cross Hospital, Okayama, Japan; 4 Department of Clinical Oncology, Kagawa University Hospital, Kita-gun, Japan; 5 Department of Gastroenterology and Hepatology, Dentistry and Pharmaceutical Sciences, Okayama University Graduate School of Medicine, Okayama, Japan; 6 Department of Chemotherapy, Yachiyo Medical Center, Tokyo Women’s Medical University, Yachiyo, Japan; 7 Cancer Treatment Center, Kansai Medical University Hospital, Hirakata, Japan; 8 Department of Digestive Surgery, Nakadori General Hospital, Akita, Japan; 9 Department of Gastroenterology and Hepatology, Kurashiki Central Hospital, Kurashiki, Japan; 10 Department of Internal Medicine, Okayama Saiseikai General Hospital, Okayama, Japan; 11 Department of Internal Medicine, Okayama Medical Center, Okayama, Japan

**Keywords:** Gastric cancer, Peripheral neuropathy, Oxaliplatin, Paclitaxel

## Abstract

**Background:**

Paclitaxel is used in second-line conventional chemotherapies to manage patients with unresectable advanced gastric cancer (GC). Paclitaxel-induced peripheral neuropathy is a known adverse event leading to treatment discontinuation. Additionally, oxaliplatin which causes irreversible peripheral neuropathy is now commonly used in first-line chemotherapy for advanced GC in Japan. Thus, examining the incidence of peripheral neuropathy with paclitaxel after oxaliplatin is necessary to improve the quality of life and outcomes of patients with advanced GC in the second-line treatment setting.

**Methods:**

This prospective observational multicenter study, (which we named IVY study), will evaluate the degree of chemotherapy-induced peripheral neuropathy (CIPN) and the efficacy of second-line chemotherapy for unresectable advanced GC. A patient neurotoxicity questionnaire (PNQ) and the Functional Assessment of Cancer Therapy/Gynecologic Oncology Group-Neurotoxicity (FACT/GOG-Ntx) will be used to assess CIPN during the second-line treatment. The key eligibility criteria are as follows: 1) unresectable or recurrent GC histologically confirmed to be primary adenocarcinoma of the stomach, 2) age over 20 years, 3) Eastern Cooperative Oncology Group performance status score of 0–2, 4) written informed consent following full study information is provided to the patient, 5) progression or intolerance for first-line chemotherapy comprising fluorinated pyrimidine and platinum anticancer drugs (cisplatin or oxaliplatin) for advanced GC. 6) presence of evaluable lesions as confirmed using a computed tomography (CT) or magnetic resonance imaging. A total of 200 patients is considered to be appropriate for inclusion in this study.

**Discussion:**

The results of this study will provide some information on CIPN with the sequential usage of oxaliplatin as first-line chemotherapy to paclitaxel as second-line chemotherapy in clinical practice.

**Trial registration:**

This trial is registered in the University Hospital Medical Information Network’s Clinical Trials Registry with the registration number UMIN000033376 (Registered 11 July 2018).

## Background

Gastric cancer (GC) is the fifth common cancer and the third common cause of cancer-related mortality worldwide [1]. Standard chemotherapy with a platinum-based chemotherapeutic and fluoropyrimidine is widely used as first-line treatment for advanced GC [2–4]. In the second-line setting, the survival benefit of cytotoxic chemotherapy using docetaxel or irinotecan was recently ascertained in several randomized trials [5–7]. Weekly administration of solvent-based (sb)-paclitaxel achieved overall survival (OS) that was similar to that with irinotecan in a phase III trial [8] and has become the control arm in several global trials [9, 10]. In the phase III RAINBOW trial, ramucirumab, an anti-vascular endothelial growth factor receptor 2 antibody, in combination with sb-paclitaxel significantly improved OS compared with sb-paclitaxel alone in patients with advanced GC after first-line platinum- and fluoropyrimidine-based chemotherapy [9]. Subsequently, in the second-line setting, ramucirumab plus sb-paclitaxel has become the most recommended regimen in the Japanese Gastric Cancer Treatment Guidelines 2018 (ver. 5).

Nanoparticle albumin-bound (nab)-paclitaxel is a solvent-free, albumin-bound 130-nm particle formulation of paclitaxel, which reduces the risk of hypersensitivity reactions caused by polyethoxylated castor oil and does not require hydrated ethanol as a solvent [11, 12]. Therefore, nab-paclitaxel can also be used in patients with alcohol intolerance. The ABSOLUTE trial demonstrated that weekly nab-paclitaxel was non-inferior to weekly sb-paclitaxel in terms of OS and achieved a better trend of overall response rate (ORR) and progression-free survival (PFS) in second-line therapy for unresectable advanced GC [13]. Additionally, in a recent Japanese phase II trial, combination therapy with nab-paclitaxel and ramucirumab showed good efficacy and manageable toxicity in patients with advanced GC refractory to first-line chemotherapy [14]. Based on these clinical trial results, in addition to the most recommended regimen of sb-paclitaxel and ramucirumab, nab-paclitaxel monotherapy and nab-paclitaxel plus ramucirumab combination therapy were frequently used as second-line treatment in recent Japanese clinical practice.

CIPN is a common treatment-related adverse event (AE) that impacts the long-term quality of life of cancer patients. CIPN can potentially cause dose modifications or early discontinuation of treatment, and there are no established agents recommended for the prevention of CIPN in patients with cancer undergoing treatment with neurotoxic agents [15]. Paclitaxel has long been acknowledged as a chemotherapeutic that can induce CIPN, which is dose-limiting and cumulative. Recent studies on weekly administration of sb-paclitaxel- or nab-paclitaxel-containing regimens in second-line therapy for unresectable advanced GC demonstrated that the estimated incidence of paclitaxel-induced CIPN (all grades based on the Common Terminology Criteria for Adverse Events [CTCAE]) was approximately 60% and that the incidence of paclitaxel-induced grade 3 or higher CIPN ranged from 2 to 8% [8, 9, 13]. In the ABSOLUTE trial, the most common adverse drug reaction leading to treatment discontinuation was peripheral neuropathy (2% in the weekly nab-paclitaxel group and 1% in the weekly sb-paclitaxel group) [13].

Recently, a randomized phase III trial of doublet therapy with S-1 and cisplatin (CS) or S-1 with oxaliplatin (SOX) in the first-line setting of advanced GC showed that oxaliplatin was as effective as cisplatin in terms of OS and PFS [16]. SOX is generally less toxic and has more clinical convenience; forced hydration is not required with SOX, unlike cisplatin. Gradually, SOX has been replacing CS in first-line treatment of advanced GC in Japan.

However, oxaliplatin, similar to paclitaxel, can lead to irreversible peripheral neuropathy. In the above-mentioned phase III trial, the incidence of sensory neuropathy in the SOX group was very high (all-grade, 85.5%; grade 3 or worse, 4.7%). Oxaliplatin-induced CIPN is dose-dependent and worst symptoms emerge 3 months after the end of administration [17]. Therefore, oxaliplatin-induced CIPN in first-line treatment might influence dose intensity and treatment duration of paclitaxel and, as a result, decrease the efficacy of paclitaxel-containing regimens in second-line treatment. Conversely, irinotecan and ramucirumab monotherapy are considered not to be influenced by oxaliplatin-induced CIPN. Past randomized phase III trials of paclitaxel in the second-line setting of advanced GC did not include patients who receive oxaliplatin as first-line treatment and/or patients with a certain level of peripheral neuropathy before the initiation of a paclitaxel-containing regimen. No studies are elucidating the relationship between CIPN and treatment efficacy in second-line chemotherapy of advanced GC.

The recent standard approach to AEs that occur during anticancer treatment is the physician-rated CTCAE, which is maintained by the US National Cancer Institute. Multiple studies reported that this physician-rated approach misses as many as 50% of all AEs compared with patient-reported outcomes (PRO) measures and that PRO measures improved the detection and precision of AE measurement [18, 19]. The rates of CIPN reported by physicians were lower than those reported by patients, and physician-rated scales exhibited substantially lower sensitivity and reliability compared to patient-reported CIPN scales [20, 21]. The US Food and Drug Administration recommended the use of PRO measures for AE measurement in oncology drug development [22]. In this prospective study, a patient neurotoxicity questionnaire (PNQ) and the Functional Assessment of Cancer Therapy/Gynecologic Oncology Group-Neurotoxicity (FACT/GOG-Ntx) will be used to assess CIPN based on patient reports, whereas the CTCAE version 4.0 will be used as the physician-rated CIPN assessment before and during second-line treatment for advanced GC. The PNQ and FACT/GOG-Ntx target symptoms and concerns associated with CIPN [23, 24]. These PRO measures contain questions designed to evaluate the severity and impact of neuropathy symptoms on people’s lives. Based on the viewpoints of both patient-reported and physician-rated assessments, we will evaluate the relationship between the degree of CIPN and the efficacy of second-line chemotherapy for unresectable advanced GC in this prospective observational multicenter study.

## Methods

### Study objectives

The primary objective of this prospective observational multicenter study is to evaluate the incidence and development of CIPN in patients with and without CIPN at the start of second-line chemotherapy for unresectable advanced GC.

### Study setting

This study is conducted in accordance with the World Medical Association Declaration of Helsinki and Japanese Ethical Guidelines for Medical and Health Research Involving Human Subjects [25]. The trial protocol has been approved by the Institutional Review Board of all participating institutions and the Kawasaki Medical School Hospital. The protocol of this study has been registered in the University Hospital Medical Information Network’s Clinical Trials Registry (registration number, UMIN000033376).

### Study design and assessment

The primary endpoint is the incidence of grade 3–4 CIPN in second-line chemotherapy. The secondary endpoints are ORR, OS, PFS, time to treatment failure (TTF), safety (the incidence of AEs), and the relationship between the degree of CIPN and the efficacy. This study blood samples in two points (before and after second-line treatment) will be collected for ancillary research to explore the biomarker of paclitaxel efficacy and CIPN.

The PNQ and FACT/GOG-Ntx, patient-reported outcome measures, will be used to assess CIPN because these are valid and reliable instruments for assessing CIPN in patients treated with taxane or oxaliplatin [20, 26, 27]. Patients will answer the PNQ and the FACT/GOG-Ntx questionnaires before treatment (baseline) and every treatment cycle. The PNQ includes two questionnaire items: one inquiring sensory neurotoxicity and one inquiring motor neurotoxicity [23]. The questionnaire items are designed to correspond with the neurotoxicity questions included in the CTCAE. The PNQ grades range from grade A (no symptom) to grade E (very severe neuropathy). Grades from A to C indicate an absence of symptoms interfering with activities of daily living, whereas grades from D and E indicate CIPN symptoms that interfere with activities of daily living. The FACT/GOG-Ntx questionnaire comprises 11 items related to neurotoxicity, with each rated on a five-point scale (0 to 4) [24]. The possible score range for the FACT/GOG-Ntx scale is from 0 to 44, with high scores indicating a lower grade of neuropathy. Tumor assessment using diagnostic imaging will be carried out every within 12 weeks (+ 2 weeks), and treatment response will be evaluated using the Response Evaluation Criteria in Solid Tumors (RECIST) ver. 1.1 [28]. PFS is defined as the time from registration to the time of progression after second-line treatment initiation or death from any cause. OS is defined as the time from registration to the time of death or last contact. The severity of AEs will be assessed using CTCAE 4.0 [29]. To investigate the influence of first-line treatment to second-line tumor response, we will collect data on treatment duration, tumor response, and total dose of platinum agents in first-line chemotherapy.

### Eligibility criteria

1. Patients with unresectable or recurrent GC histologically confirmed as primary adenocarcinoma of the stomach
2. Patients aged over 20 years
3. Patients with an Eastern Cooperative Oncology Group performance status score of 0–2
4. Patients who have been fully informed of this study and provided written informed consent
5. Patients with progression or intolerance for first-line chemotherapy comprising fluorinated pyrimidine anticancer drugs (e.g., 5-fluorouracil, S-1, capecitabine, UFT) and platinum anticancer drugs (cisplatin or oxaliplatin) for unresectable or recurrent GC
6. Presence of evaluable lesions as confirmed using a computed tomography (CT) or magnetic resonance imaging

### Exclusion criteria

1. Patients with a life expectancy of shorter than 3 months
2. Patients with severe complications (angina pectoris, myocardial infarction, or arrhythmia) or uncontrollable diabetes mellitus, blood hypertension, or bleeding tendency
3. Patients with a history of serious allergic reactions or serious drug allergy
4. Patients with a clinically relevant mental disorder that prohibits response to questionnaires
5. Patients for whom the attending physician considered that enrollment in the study is inappropriate

### Treatment methods

**Fig. 1:**
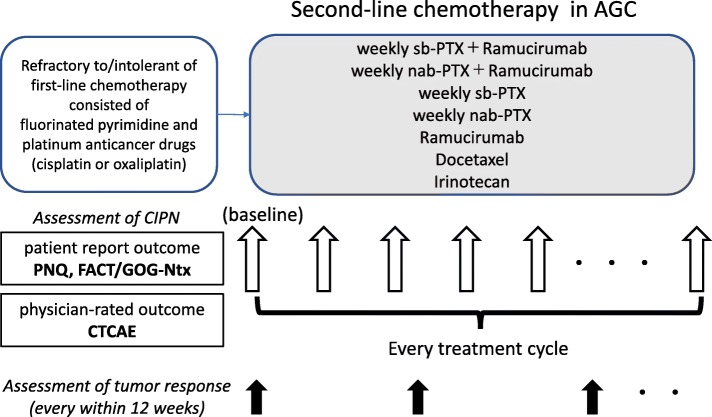
IVY study design. Patients will answer the PNQ and the FACT/GOG-Ntx questionnaires before treatment (baseline) and every treatment cycle

All recommended regimens in the Japanese Gastric Cancer Treatment guidelines 2018 (ver. 5) and the Pan-Asian adapted European Society for Medical Oncology Clinical Practice Guidelines will be allowed in this study [30]. Each physician will be able to select the appropriate regimen with consideration of each patient’s conditions (Fig. 1). The definitive regimens are as follows. Sb-paclitaxel plus ramucirumab regimen will comprise ramucirumab (80 mg/m2 intravenously on days 1 and 15) with sb-paclitaxel (80 mg/m2 intravenously on days 1, 8, and 15) every 4 weeks. Nab-paclitaxel plus ramucirumab regimen will comprise ramucirumab (80 mg/m2 intravenously on days 1 and 15) with nab-paclitaxel (100 mg/m2 intravenously on days 1, 8, and 15) every 4 weeks. Weekly sb-paclitaxel (80 mg/m2) will be administered intravenously on days 1, 8, and 15, every 4 weeks. Weekly nab-paclitaxel (100 mg/m2) will be administered intravenously on days 1, 8, and 15, every 4 weeks. Ramucirumab (8 mg/kg) will be administered intravenously on days 1 and 15, every 4 weeks. Docetaxel (60–70 mg/m2) will be administered intravenously on day 1, every 4 weeks. Irinotecan (150 mg/m2) will be administered intravenously on days 1 and 15, every 4 weeks. Dose reduction and/or cycle delays will be permitted according to the decision of each physician.

### Statistical methods

As mentioned in the background section, of the patients who enrolled in this study, the patients with or without any degree of CIPN will be estimated 1: 2 population at the start of second-line chemotherapy administration. We estimate the incidence of grade 3–4 CIPN with 8% (SD + 8%) of the enrolled patients without CIPN at the start of second-line chemotherapy administration during the second-line treatment of PTX with Ramucirumab group (control group). Next, hypothetically, patients with CIPN with any grade at the start of second-line chemotherapy administration (test group) will increase the incidence of grade 3–4 CIPN by + 5% during second-line treatment. To confirm the difference of the incidence of grade3–4 CIPN between the two groups with verifying with α = 0.05 (both sides) and the power (1 − β) = 0.8, the sample size is calculated to be 83 cases in total. The participation ratio is 1: 2 for patients with and without CIPN at the start of second-line chemotherapy, resulting in a total sample size of 125. Among patients scheduled to participate in this study, assuming that 70% of participants will receive the standard treatment of PTX with ramucirumab, the target sample size will be 179. The number of cases for a recruit is set to 200, taking into consideration the participation of incorrect cases and cases of dropout. The degree and frequency of CIPN were evaluated by the PNQ, FACT/GOG-Ntx, and CTCAE. PFS will be estimated by the Kaplan-Meier method and compared among groups with the stratified log-rank test. Secondary endpoints are the rate of AEs graded according to the CTCAE version 4.0, ORR according to the RECIST version 1.1., PFS, and OS. Categorical data comparisons according to the degree and frequency of CIPN will be performed using Fisher’s exact and the χ2 tests. To assess the correlation between the PNQ, FACT/GOG-Ntx questionnaires and the physician-rated CTCAE scales, Spearman’s correlation coefficient was utilized for this evaluation.

### Follow-up

Disease progression and occurrence of metastasis, synchronous, or metachronous cancer will be monitored by abdominal computed tomography, magnetic resonance imaging, evaluation of increased clinical symptoms, or elevated levels of tumor markers such as carcinoembryonic antigen, carbohydrate antigen (CA) 19–9, and CA125, every 12 weeks during the treatment period. Safety will be assessed by monitoring AEs using physical and laboratory examinations. The survey sheets, including those of safety, efficacy, and compliance with treatment, will be collected at the time of registration and after every treatment cycle. Besides, patient outcomes will be investigated 2 years after study initiation as well as 1 year after the accrual of the last patient. The CIPN assessments will be performed at baseline and before every cycle using the PNQ, FACT/GOG-Ntx, and CTCAE during the treatment period.

## Discussion

In the first-line setting of advanced GC, a randomized phase III trial of doublet therapy with CS or SOX showed that oxaliplatin was as effective as cisplatin concerning OS and PFS [16]. The Pan-Asian adapted European Society for Medical Oncology Clinical Practice Guidelines recommend doublet platinum/fluoropyrimidine combinations for fit patients with advanced GC and state that oxaliplatin is the preferred option due to its favorable safety profile and ease of administration [30]. Gradually, in Japan, SOX has been replacing CS in the first-line treatment of advanced GC. Oxaliplatin-induced CIPN is characterized by dose-dependent symptoms that worsen after the end of treatment [17]. Therefore, in some cases, oxaliplatin in first-line treatment can potentially reduce the efficacy of paclitaxel-containing regimens in second-line treatment. However, it remains unclear whether the degree of peripheral neuropathy with paclitaxel after oxaliplatin influences the efficacy of paclitaxel-containing regimens in second-line treatment.

In this study, we plan to compare the efficacy of paclitaxel-containing regimens in second-line treatment by assessing the rate of remaining CIPN not only immediately before the administration of paclitaxel but also between patients receiving cisplatin or oxaliplatin in first-line treatment.

To evaluate CIPN, we will use the PNQ and the FACT/GOG-Ntx as patient-reported outcomes and the CTCAE version 4.0 as the physician-rated outcome. If this study reveals that the PNQ and the FACT/GOG-Ntx can detect the patients who are fate to have over Grade 3 CIPN earlier than the CTCAE in patients receiving paclitaxel-containing regimens, this result will implicate daily usage of the PNQ and the FACT/GOG-Ntx may provide clinical benefit to patients by predicting severe CIPN before onset. In addition, the results of this study will provide some indication on the influence of CIPN by the practical use of oxaliplatin in first-line treatment on the efficacy of second-line chemotherapy for unresectable advanced GC in the near future.

## Additional file

**Additional file 1.** Name of the ethics committees and Committee’s reference number

## Data Availability

Not applicable.

## Abbreviations

AE: Adverse event
CA: Carbohydrate antigen
CIPN: Chemotherapy-induced peripheral neuropathy
CS: S-1 and cisplatin
CTCAE: Common Terminology Criteria for Adverse Events
FACT/GOG-Ntx: Functional Assessment of Cancer Therapy/Gynecologic Oncology Group-Neurotoxicity
GC: Gastric cancer
nab: Nanoparticle albumin-bound
ORR: Objective response rate
OS: Overall survival
PFS: Progression-free survival
PNQ: Patient Neurotoxicity Questionnaire
PRO: Patient-reported outcome
RECIST: Response Evaluation Criteria in Solid Tumors
sb: Solvent-based
SOX: S-1 and oxaliplatin
TTF: Time to treatment failure
