# Supplementary material for: The relationship between peripheral neuropathy and efficacy in second-line chemotherapy for unresectable advanced gastric cancer: a prospective observational multicenter study protocol (IVY)
Source: BMC Cancer. 2019 Oct 11;19:941. doi: 10.1186/s12885-019-6163-6 (PMC6788076; doi:10.1186/s12885-019-6163-6)
Supplement: Supplementary file 1 — Additional file 1. Name of the ethics committees and Committee’s reference number [file 12885_2019_6163_MOESM1_ESM.docx]

 Name of the ethics committees:  Committee’s reference number

Research Ethics Committee of Kawasaki Medical School and Hospital: 3207

Ethics Committee of Tokyo Women's Medical University: 5030

Ethics Committee of Okayama Rosai Hospital: 151

Ethics Committee of Japan Community Healthcare Organization, Kyushu Hospital: 590

Ethics Committee of Konko Hospital: H31-1

Research Ethics Committee of Nakadori General Hospital: 237

Okayama University Graduate School of Medicine, Dentistry and Pharmaceutical Sciences and Okayama University Hospital, Ethics Committee: 1812-017

Institutional Review Board of Okayama Saiseikai General Hospital: 190101

The research ethics committee of Aizawa Hospital: 2018-057

Ethics Committee of Japanese Red Cross Okayama Hospital: H30-62

Medical Ethics Committee of Kurashiki Central Hospital: 2959

Ethics Committee of Ishikawa Prefectural Central Hospital Department of Clinical Oncology: 1214

Research Ethics Comittee (REC)of Steel Memorial Muroran Hospital: J181004

Okayama Medical Center, Research Ethics Committee: 2018-140/ 2019-083

Ethics Review Board, University of Toyama: 30-175
